# Altered myocardial response in patients with diabetic retinopathy: an exercise echocardiography study

**DOI:** 10.1186/s12933-015-0281-5

**Published:** 2015-09-18

**Authors:** Zhe Zhen, Yan Chen, Kendrick Shih, Ju-Hua Liu, Michele Yuen, David Sai-Hung Wong, Karen Siu-Ling Lam, Hung-Fat Tse, Kai-Hang Yiu

**Affiliations:** Division of Cardiology, Department of Medicine, Queen Mary Hospital, The University of Hong Kong, Rm 1929b, Block K, Hong Kong, China; Department of Ophthalmology, Queen Mary Hospital, The University of Hong Kong, Hong Kong, China; Division of Endocrinology, Department of Medicine, Queen Mary Hospital, The University of Hong Kong, Hong Kong, China; Research Centre of Heart, Brain, Hormone and Healthy Aging, Li Ka Shing Faculty of Medicine, The University of Hong Kong, Hong Kong, China; Department of Medicine, The University of Hong Kong ShenZhen Hospital, Shenzhen, China

**Keywords:** Diabetic cardiomyopathy, Cardiac dysfunction, Exercise echocardiography, Diabetic retinopathy, Cardiac function reserve

## Abstract

**Background:**

Type 2 diabetes mellitus (T2DM) complicated by retinopathy is associated with altered left ventricular (LV) structure and resting myocardial dysfunction unlike T2DM without retinopathy. The myocardial response to stress has not been compared in patients with and without diabetic retinopathy. The aim of this retrospective study was to determine the relationship between retinopathy and myocardial function in patients with T2DM at rest and during exercise echocardiography.

**Methods:**

134 patients with T2DM and no evidence of underlying coronary artery disease were recruited. All patients underwent retinal photography to screen for diabetic retinopathy, and resting and exercise echocardiography. Resting echocardiography was analyzed by conventional echocardiographic parameters and speckle tracking derived global longitudinal strain (GLS). Exercise echocardiography parameters included diastolic function reserve index (DFRI) and stress GLS.

**Results:**

The mean age of participants was 60 years and 49 % were male. Diabetic retinopathy was identified in 43 patients (32 %). Resting echocardiography revealed that those with diabetic retinopathy had a higher prevalence of impaired diastolic function, higher E/E′ ratio (LV filling pressures) and impaired resting GLS compared with those without. Exercise echocardiography revealed that those with diabetic retinopathy also had more impaired DFRI and stress GLS. Multivariable analysis showed that the presence of diabetic retinopathy was independently associated with high resting E/E′, diastolic dysfunction grade, impaired resting GLS, low DFRI and impaired stress GLS.

**Conclusions:**

In conclusion, the presence of diabetic retinopathy was independently associated with impaired resting myocardial function (diastolic and systolic function) and myocardial function during stress (evaluated by DFRI and stress GLS).

**Electronic supplementary material:**

The online version of this article (doi:10.1186/s12933-015-0281-5) contains supplementary material, which is available to authorized users.

## Background

Patients with type 2 diabetes mellitus (T2DM) have altered left ventricular (LV) structure and function, independent of traditional risk factors and underlying coronary artery disease, that leads to an adverse cardiovascular outcome [1, 2]. One of the proposed pathologies that contributes to the adverse change to the myocardium is diabetes-induced microvascular dysfunction [3]. Cardiac magnetic resonance imaging has determined that the presence of diabetic retinopathy, a manifestation of underlying microvascular dysfunction, is associated with increased LV concentric remodeling [4]. Further, resting echocardiography has demonstrated that patients with more severe diabetic retinopathy have a greater left ventricular mass and decreased LV ejection fraction [5]. Although resting echocardiography provides information about myocardial function at a static state, the dynamic response of the myocardium to stress can only be revealed by stress testing [6]. Patients with T2DM have impaired LV function during pharmacological or exercise stress that is not present at rest [7, 8]. Stress echocardiography-derived LV functional reserve has been shown to predict adverse outcome in patients with T2DM better than resting echocardiography parameters [9]. Nonetheless the myocardial response during stress has not been compared between patients with and without diabetic retinopathy. The aim of this study was to determine the relationship between retinopathy and myocardial function in patients with T2DM at rest and during a stress state by exercise echocardiography.

## Methods

A total of 323 patients with T2DM, as defined by World Health Organization criteria, were consecutively recruited at Queen Mary Hospital, Hong Kong [10]. Patients with a documented history of cardiovascular disease, including coronary artery disease, myocardial infarction, significant valvular disease, stroke or peripheral vascular disease, were excluded to eliminate their potential confounding risk of impaired cardiac function (n = 78) [11]. The remaining 245 patients were recruited for the present retrospective study. Written informed consent was obtained from all participants. The study was approved by the local institutional review board of the Hong Kong West Cluster and was conducted according to the Declaration of Helsinki. This study is part of the Chinese Diabetic Heart Study (CDATS) to evaluate cardiovascular manifestations in Chinese patients with T2DM, in an attempt to evaluate the pathophysiology and potential therapies [12].

All patients underwent a complete physical examination and an interview to establish baseline characteristics. Smoking status was recorded as positive if patients currently or had ever smoked. Body-mass index (BMI) was calculated in kg/m^2^ and an electrocardiogram (ECG) recorded. Hypertension was defined as resting systolic or diastolic blood pressure ≥140/90 mmHg at two different clinic visits or prescription of antihypertensive medication. Data on medications were retrieved from patients’ electronic medical record. A fasting blood sample was obtained to measure HbA1c, fasting plasma glucose, total cholesterol, triglyceride, high-density lipoprotein cholesterol (HDL), low-density lipoprotein cholesterol (LDL) and serum creatinine level. The estimated glomerular filtration rate (eGFR) was calculated using the Modified Diet in Renal Disease Equation [13].

For diabetic retinopathy screening, digital photographs of the retina covering two standard fields, focused on the optic disc and macula respectively, were taken by an optometrist using a non-mydriatic fundus camera (Topcon TRC-50DX: Type IA). The photographs were then graded by both the optometrist and an ophthalmologist at the Department of Ophthalmology, University of Hong Kong, using the UK National Screening Program for Diabetic Retinopathy Grading Scheme: no DR (R0), background (non-proliferative) DR (R1), pre-proliferative DR (R2), proliferative DR (R3), maculopathy (M1) and photocoagulation (P) [14]. Any discrepancy between graders was arbitrated by a senior ophthalmologist. Patients found to have at least background DR (R1) on fundus photography were referred to the specialist ophthalmology clinic at Queen Mary Hospital, Hong Kong for a complete eye examination, including a dilated fundus examination for confirmation. Patients defined as having retinopathy had at least background retinopathy.

Transthoracic echocardiographic examination was performed in all patients using a commercially available echocardiography system (Vingmed Vivid 7, General Electric Vingmed Ultrasound, Milwaukee, WI, USA) with the patient lying in the lateral decubitus position. A 3.5-MHz transducer was used to obtain images that were digitally stored in cine-loop format. The frame rate was set at > 90 frames per second. Off-line analysis was performed using the EchoPAC version 108.1.5 (General Electric Vingmed, Horten, Norway). All patients were in normal sinus rhythm during the examination.

The inter-ventricular septum thickness, posterior wall thickness, and LV dimension were measured in M-mode according to the current recommendations. LV volume and ejection fraction were determined from apical four and two-chamber views using the modified Simpson’s biplane method of discs. Evaluation of LV diastolic function was based on the pulsed-wave Doppler of mitral valve inflow. Peak velocity in early diastole (E-wave) and late diastole (A-wave) was measured and the E/A ratio calculated. Pulsed wave tissue Doppler imaging was used to measure the early diastolic velocity (E′) with the sample volume placed at the septum annulus. In addition, the E/E′ ratio was calculated as an estimation of LV filling pressure [15]. LV mass index (LVMi) was calculated by Devereux’s formula and indexed to body surface area [16]. Diastolic function at rest was classified as previously described: normal; Grade 1, defined as LV impaired relaxation without evidence of increased filling pressure; Grade 2, LV impaired relaxation associated with moderate elevation of filling pressure or pseudo-normal filling; or Grade 3, restrictive LV filling [17]. Tricuspid regurgitation jet velocity was also obtained using continuous-wave Doppler imaging to estimate right ventricle systolic pressure (RVSP).

The measurement of LV global longitudinal stain (GLS) provides detailed assessment of LV myocardial deformation by tracking natural acoustic markers in a frame-to-frame basis within the cardiac cycle [18]. In brief, longitudinal strain, assessing the shortening/lengthening of the myocardial wall, was measured from 3 apical views: 2-chamber view (comprising anterior and inferior walls), 4- chamber view (posteroseptal and lateral walls) and 3-chamber view (anteroseptal and posterior wall). Each wall was subsequently divided into 3 areas (basal, mid and apical) and a total of 18 segmental strain curves were obtained. During measurement, the regions of interest were manually adjusted to include the entire myocardial thickness while avoiding simultaneous inclusion of the pericardium in the same area [19] GLS was calculated as the average value of the three apical strain peak values. The intra and inter-observer reliability of strain analysis by our group has been previously reported [20].

Of the recruited patients, 55 of the 245 were physically unable to perform exercise echocardiography. Exercise echocardiography was performed in the remaining 190 patients. Regional wall motion abnormality was detected in 22 patients during stress testing, suggesting the presence of significant ischemic heart disease and 34 patients had suboptimal stress images for analysis. The number of patients with exercise echocardiography images suitable for analysis was thus 134. The mean age of the remaining participants was 60 years and 49 % were male. Diabetic retinopathy was indicated in 43 patients (32 %). Maximum exercise treadmill testing was performed using the standard Bruce protocol [21]. All parameters measured at rest were obtained again within 2 min of exercise completion. It has been shown that the primary mechanism for the increase in E′ during exercise may be increased sympathetic tone and consequent faster myocardial relaxation. Based on the findings, an LV diastolic function reserve index (DFRI) was calculated by the formula: DFRI = ∆E′ × E′ base (∆E′ is the change of E′ from resting state to the end of exercise. E′ base is the early diastolic mitral annular velocity at rest) [22].

All continuous variables and categorical variables are expressed as mean ± standard deviation and frequencies or percentages, respectively. Continuous variables were compared between patients with and without diabetic retinopathy, using independent Student’s *t* test. Categorical variables were compared using Chi square test or Fisher’s exact test if >10 % of the cells had an expected count <5. Pearson’s correlation test was used to assess the correlation of retinopathy with myocardial function in diabetic patients. Correlation coefficients were performed in order to assess the association of METS and resting and exercise echocardiography parameters in T2DM patients. Multivariate analyses were performed to detect the independent predictors of abnormal myocardial function in patients with T2DM. In these analyses, factors independently associated with E/E′, LV diastolic dysfunction grade, resting GLS, DFRI or stress GLS were identified using an enter regression model in which each statistically significant univariate predictor of outcomes with p < 0.10 was chosen. All data analysis was performed using the commercially available statistics software SPSS for Windows (Version 17.0). All p values reported are 2-sided for consistency. A p value < 0.05 was considered statistically significant.

## Results

The baseline characteristics of patients with and without diabetic retinopathy are shown in Table 1. Age, hyperlipidemia, smoking status and BMI were similar in both groups of patients. Serum levels of triglyceride, total cholesterol, HDL, LDL, fasting glucose, HbA1c were also comparable. Patients with diabetic retinopathy were more likely to be female, have longer disease duration, and be hypertensive and prescribed insulin (Table 1).

**Table 1:** Baseline clinical characteristics in patients with and without retinopathy

Parameters	No retinopathy	Retinopathy	P
	N = 91	N = 43	
Demographic information			
Age (year)	59.7 ± 9.4	60.8 ± 9.1	0.52
Male, n (%)	50 (55 %)	15 (35 %)	0.03
Disease duration (years)	13.6 ± 7.4	17.9 ± 7.5	<0.01
Hypertension, n (%)	65 (71 %)	36 (88 %)	0.04
Hyperlipidemia, n (%)	45 (50 %)	25 (60 %)	0.31
Smoking, n (%)	21 (24 %)	6 (15 %)	0.27
Body mass index (kg m^−2^)	26.8 ± 5.3	26.2 ± 4.0	0.54
Blood biochemistry			
Triglyceride (mmol L^−1^)	4.1 ± 1.1	4.2 ± 1.0	0.59
Total cholesterol (mmol L^−1^)	1.8 ± 1.4	1.7 ± 1.3	0.78
High-density lipoprotein (mmol L^−1^)	1.3 ± 0.4	1.2 ± 0.4	0.31
Low-density lipoprotein (mmol L^−1^)	2.4 ± 0.6	2.3 ± 0.7	0.57
eGFR (mL/min/1.73 m^2^)	88.7 ± 21.6	113.9 ± 202.1	0.25
HbA1c (%)	7.5 ± 1.3	8.0 ± 1.4	0.06
Medication, n (%)			
Insulin	38 (44 %)	29 (69 %)	<0.01
Diuretics	7 (8 %)	4 (10 %)	0.76
ACEI/ARB	50 (56.8 %)	23 (54.8 %)	0.83

*ARB* angiotensin receptor blocker, *ACEI* angiotensin converting enzyme inhibitors, *eGFR* estimated glomerular filtration rate

Conventional echocardiographic LV findings are described in Table 2. Resting LV dimension, LV mass index and LV ejection fraction were similar between patients with and without diabetic retinopathy. In addition, all patients had preserved systolic function with an LV ejection fraction ≥55 %. Nonetheless patients with diabetic retinopathy had a higher prevalence of impaired diastolic function and a higher E/E′ ratio as well as impaired LV systolic function as reflected by a lower resting GLS.

**Table 2:** Conventional echocardiographic parameters in patients with and without retinopathy

	No retinopathy	Retinopathy	P
	N = 91	N = 43	
LVDd (mm)	44.7 ± 5.1	43.6 ± 5.1	0.25
LVDs (mm)	28.9 ± 3.7	28.1 ± 3.5	0.23
IVSd (mm)	11.3 ± 2.2	12.0 ± 1.6	0.06
IVSs (mm)	14.2 ± 2.4	14.8 ± 3.6	0.26
LVPWd (mm)	11.0 ± 1.5	11.1 ± 1.5	0.79
LVPWs (mm)	14.7 ± 2.0	14.3 ± 2.0	0.34
LV mass index (g)	211 ± 66	212 ± 60	0.93
LV ejection fraction (%)	59.7 ± 5.0	58.1 ± 6.0	0.12
E (m/s)	0.8 ± 0.2	0.7 ± 0.2	0.39
E/A ratio	0.94 ± 0.26	0.89 ± 0.55	0.45
E′ (m/s)	0.10 ± 0.02	0.09 ± 0.03	<0.01
E/E′ ratio	7.5 ± 2.4	8.8 ± 3.6	0.02
Diastolic function grade, n (%)			<0.01
Normal	52 (58 %)	10 (23 %)	
Grade1	24 (27 %)	26 (61 %)	
Grade2	11 (12 %)	6 (14 %)	
Grade3	2 (2.2 %)	1 (2.33 %)	
GLS (%)	−18.3 ± 2.0	−17.2 ± 2.3	<0.01

*E* trans-mitral early diastolic peak velocity, *E′* septal annulus early diastolic peak velocity, *GLS* global longitudinal strain, *IVSd* interventricular septa thickness at end-diastole, *IVSs* interventricular septal systolic thickness at end-systole, *LVDd* left ventricle dimension at end-diastole, *LVDs* left ventricle dimension at end-systole, *LVPWd* left ventricle posterior wall thickness at end-diastole, *LVPWs* left ventricle posterior wall thickness at end-systole

Exercise echocardiography parameters are shown in Table 3. Patients with retinopathy had lower exercise LV ejection fraction and higher E/E′ ratio compared with those without retinopathy, but not its Δ, compared with those without retinopathy. The EA ratio at rest and during stress was comparable for the two groups. Importantly, DFRI and both stress and ∆ of GLS were more impaired in patients with retinopathy.

**Table 3:** Exercise echocardiography and treadmill parameters in patients with and without retinopathy

Parameters	No retinopathy	Retinopathy	P value
	N = 91	N = 43	
Exercise echocardiography			
LVEF (%)			
Rest	59.7 ± 5.0	58.1 ± 6.0	0.12
Exercise	64.5 ± 6.3	61.9 ± 6.6	0.03
∆	4.9 ± 7.0	3.8 ± 7.3	0.41
E/A ratio			
Rest	0.94 ± 0.26	0.89 ± 0.55	0.45
Exercise	1.01 ± 0.37	0.93 ± 0.50	0.33
∆	0.07 ± 0.43	0.04 ± 0.73	0.78
E/E′ ratio			
Rest	7.5 ± 2.4	8.8 ± 3.6	0.02
Exercise	7.8 ± 2.2	9.7 ± 4.9	<0.01
∆	0.3 ± 2.3	1.0 ± 4.1	0.27
GLS (%)			
Rest	−18.3 ± 2.0	−17.2 ± 2.3	<0.01
Exercise	−19.7 ± 2.3	−17.5 ± 2.5	<0.01
∆	−1.4 ± 1.8	−0.3 ± 2.0	<0.01
DFRI	35.2 ± 22.0	22.8 ± 19.2	<0.01
Treadmill parameters			
HR rest	82.3 ± 12.8	86.3 ± 14.2	0.10
HR exercise	154.1 ± 16.8	146.4 ± 21.0	0.02
HR reserve (bmp)	71.9 ± 17.5	61.5 ± 17.6	<0.01
HR reserve (%)	91.1 ± 31.2	73.6 ± 24.6	<0.01
METs	8.3 ± 2.9	7.1 ± 2.7	0.03

Data are expressed as mean ± SD or n (%)

*DFRI* diastolic function reserve index, *HR* heart rate, *METs* metabolic equivalents of task

Functional parameters derived from treadmill testing are shown in Table 3. Resting HR was similar for both groups but peak exercise HR, HR reserve, HR reserve % and METS achieved was significantly lower in patients with diabetic retinopathy.

**Fig. 1:**
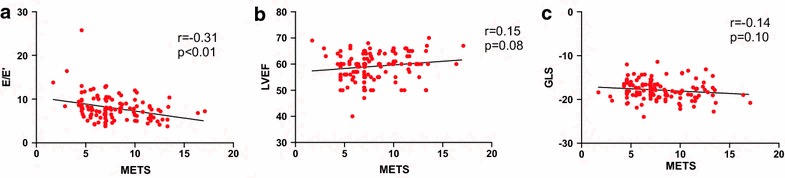
Correlation of METS with resting echocardiography parameters: **a** E/E′; **b** Left ventricular ejection fraction (LVEF); **c** Global longitudinal strain (GLS)

**Fig. 2:**
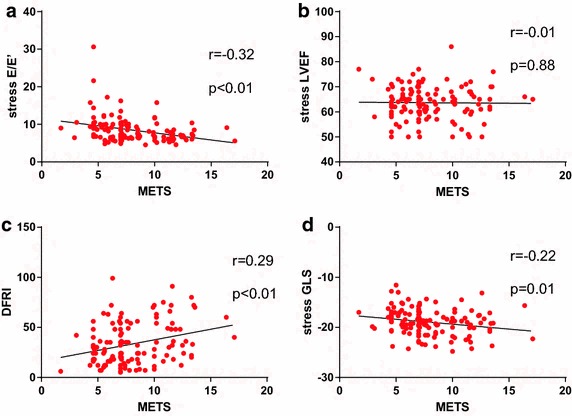
Correlation of METS with exercise echocardiography parameters: **a** Stress E/E′; **b** Stress Left ventricular ejection fraction (LVEF); **c** Diastolic functional reserve index (DFRI); **d** Stress global longitudinal strain (GLS)

The correlation of functional capacity assessed by METS with both resting and stress echocardiography parameters is shown in Figs. 1 and 2, respectively. A lower METS was significantly correlated with a higher resting E/E` ratio (Fig. 1) and a higher stress E/E′ ratio, a lower DFRI and a more impaired stress GLS (Fig. 2). Such an association was not found with LV ejection fraction and resting GLS.

Multivariable analysis adjusting for potential confounding factors of (1) resting parameters (E/E′, diastolic dysfunction grading, GLS) and (2) stress parameters (DFRI and stress GLS) is shown in Additional file 1: Tables S1–S5. The presence of diabetic retinopathy was independently associated with a high E/E′, diastolic dysfunction grade, an impaired resting GLS, a low DFRI and an impaired stress GLS.

## Discussion

The present study demonstrates that patients with T2DM complicated by retinopathy had impaired resting diastolic function and systolic function evaluated by 2D speckle tracking derived GLS. Exercise echocardiography revealed that they also had an altered myocardial stress response as demonstrated by an impaired DFRI, stress GLS and a lesser ∆ of GLS. Importantly, multivariate analysis further demonstrated that the presence of diabetic retinopathy was independently associated with both resting (resting diastolic dysfunction grading and GLS) and exercise (stress DFRI and GLS) echocardiography parameters.

### Retinopathy associated with resting myocardial structural and functional abnormality

Patients with T2DM complicated by retinopathy is common and is closely associated with glycemic control [23–25]. In addition, the presence of retinopathy has been shown to have adverse effects on LV remodeling and myocardial function that may contribute to an increased incidence of heart failure [26]. In the Multi-Ethnic Study of Atherosclerosis (MESA), narrowing of the retinal arteriolar caliber was associated with LV concentric hypertrophy as determined by cardiac magnetic resonance imaging [4]. A study of 531 patients with T2DM demonstrated that those with more severe retinopathy had increased LV mass and left atrial dimension when assessed by echocardiography [5]. In addition, LV ejection fraction was also more impaired with increasing severity of retinopathy. The present study confirmed these findings: diabetic retinopathy was associated with impaired resting diastolic function grading and systolic function measured by advanced 2D speckle tracking derived strain analysis. Collectively, these data suggest that microvascular disease may be one of the contributing factors to resting myocardial dysfunction in patients with T2DM, although the exact mechanisms are uncertain.

### Retinopathy associated with stress induced myocardial dysfunction

Myocardial performance during stress is clinically important and has been shown to be associated with hospitalization for heart failure [6]. In particular, patients with T2DM have an impaired myocardial response during stress that may subsequently affect clinical performance [27]. Recent advances in echocardiographic techniques, including tissue-Doppler imaging and speckle tracking derived strain analysis, enable detailed assessment of myocardial function during stress testing. A study of 53 T2DM patients demonstrated reduced systolic and diastolic velocity of the mitral annulus compared with controls during exercise but not at rest [7]. Using dobutamine as an inotropic agent, another study showed an impaired LV strain and strain rate during pharmacological stress in patients with T2DM compared with controls [8]. Although patients with diabetic retinopathy have adverse LV remodeling and impaired LV function at rest, the myocardial response during stress has not been studied. The present study demonstrated that patients with diabetic retinopathy had impaired diastolic function (DFRI) and LV systolic function (stress GLS) during exercise stress testing. Few hypotheses can explain the association between diabetic retinopathy and an abnormal myocardial stress response. The presence of diabetic retinopathy indicates systemic microvascular dysfunction that will likewise involve the myocardium. Typically, stress-induced wall motion abnormalities are absent in patients with microvascular disease compared with normal controls, despite a reduction in coronary flow reserve. Indeed, prior studies have consistently demonstrated that microvascular dysfunction is associated with impaired exercise capacity in patients with heart failure [28]. Collectively, microvascular disease may impair myocardial metabolism during stress, leading to an impaired response to stress, as reflected by impaired DFRI and stress GLS in patients with diabetic retinopathy. The impaired resting and stress LV function may therefore be associated with a higher incidence of heart failure in patients with diabetic retinopathy [29].

### Stress induced myocardial dysfunction and impaired functional capacity

Patients with T2DM have impaired exercise capacity due to a variety of reasons, including a blunted response in heart rate and blood pressure and autonomic dysfunction [30]. A study of 19 patients with T2DM demonstrated that resting LV diastolic dysfunction limited exercise performance, a finding confirmed by this study in which a positive correlation was established between diastolic function as measured by E/E′ ratio and METS [31]. In addition, the present study extended these findings by showing that impaired exercise diastolic (DFRI) and systolic function (stress GLS) correlated closely with exercise capacity as measured by METS. This provides evidence that impaired myocardial function during stress contributes to decreased exercise capacity in patients with T2DM. Measures that can reduce stress-induced myocardial dysfunction may thus improve exercise capacity in patients with diabetic retinopathy.

### Limitation

The present study was a cross-sectional analysis and adverse clinical outcome data were not available. The stress echocardiography parameters have nonetheless been shown to be associated with adverse outcome in patients with T2DM [9]. Future evaluation of clinical outcome should be performed to verify the clinical significance of the present findings. Further, diabetic retinopathy was not sub-classified according to its severity due to the limited study sample. A detailed assessment of myocardial response during stress in patients with varying stages of diabetic retinopathy should be evaluated within a larger population.

## Conclusion

The present study demonstrates that in patients with T2DM, the presence of retinopathy was independently associated with impaired resting and stress diastolic and systolic function. This finding suggests that patients with diabetic retinopathy have an impaired myocardial response during stress that may subsequently affect their exercise capacity and functional outcome compared with those without retinopathy.

## Additional file

Additional file 1. Multivariable analysis for individual echocardiography parameters.

## Abbreviations

A: trans-mitral late diastolic peak velocity
ACEI: angiotensin converting enzyme inhibitors
ARB: angiotensin receptor blocker
BMI: body mass index
DFRI: diastolic function reserve index
E: trans-mitral early diastolic peak velocity
E′: septal annulus early diastolic peak velocity
ECG: electrocardiogram
eGFR: estimated glomerular filtration rate
GLS: global longitudinal strain
HDL: high-density lipoprotein cholesterol
IVSd: interventricular septa thickness at end-diastole
IVSs: interventricular septal systolic thickness at end-systole
LDL: low-density cholesterol
LV: left ventricular
LVDd: LV dimension at end-diastole
LVDs: LV dimension at end-systole
LVMi: LV mass index
LVPWd: LV posterior wall thickness at end-diastole
LVPWs: LV posterior wall thickness at end-systole
METS: metabolic equivalents of task
RVSP: right ventricular systolic pressure
T2DM: type 2 diabetes mellitus
