# Supplementary material for: Altered myocardial response in patients with diabetic retinopathy: an exercise echocardiography study
Source: Cardiovasc Diabetol. 2015 Sep 18;14:123. doi: 10.1186/s12933-015-0281-5 (PMC4574544; doi:10.1186/s12933-015-0281-5)
Supplement: Supplementary file 1 — Additional file 1. Multivariable analysis for individual echocardiography parameters. [file 12933_2015_281_MOESM1_ESM.docx]

Table S1. Multivariable analysis for resting E/E’.

| Variables |  | Univariate |  |  |  | Multivariate |  |
| --- | --- | --- | --- | --- | --- | --- | --- |
|  | B | 95% CI | P |  | B | 95% CI | P |
| Age | 0.08 | 0.02-0.13 | <0.01 |  | 0.07 | 0.01-0.13 | 0.02 |
| Male | -0.82 | -1.85-0.20 | 0.12 |  |  |  |  |
| Body mass index | 0.12 | 0.02-0.22 | 0.02 |  | 0.13 | 0.03-0.23 | 0.01 |
| Smoking status | 0.35 | -0.98-1.68 | 0.60 |  |  |  |  |
| Hypertension status | 1.06 | -0.13-2.25 | 0.08 |  | 0.22 | -1.01-1.45 | 0.73 |
| Diabetic duration | 0.05 | -0.02-0.12 | 0.17 |  |  |  |  |
| Retinopathy | 1.30 | 0.22-2.37 | 0.02 |  | 1.14 | 0.06—2.22 | 0.04 |
| Insulin | 0.04 | -1.03-1.12 | 0.94 |  |  |  |  |
| Diuretics | 1.76 | -0.15-3.66 | 0.07 |  | 1.23 | -0.60-3.01 | 0.18 |
| ACEI /ARB | 0.42 | -0.66-1.49 | 0.44 |  |  |  |  |
| Triglycerides | 0.32 | -0.18-0.82 | 0.21 |  |  |  |  |
| Total Cholesterol | 0.34 | -0.06-0.74 | 0.10 |  |  |  |  |
| High-density lipoprotein | 0.37 | -0.10-1.73 | 0.60 |  |  |  |  |
| Low-density lipoprotein | 0.44 | -0.46-1.35 | 0.33 |  |  |  |  |
| HbA1c | -0.13 | -0.53-0.27 | 0.54 |  |  |  |  |

Abbreviations: ARB, angiotensin receptor blocker; ACEI, Angiotensin converting enzyme inhibitors.

Table S2. Multivariable analysis for resting diastolic dysfunction.

| Variables |  | Univariate |  |  |  | Multivariate |  |
| --- | --- | --- | --- | --- | --- | --- | --- |
|  | B | 95% CI | P |  | B | 95% CI | P |
| Age | 0.06 | 1.02-1.10 | <0.01 |  | 0.06 | 1.02-1.11 | 0.01 |
| Male | -0.48 | 0.31-1.23 | 0.17 |  |  |  |  |
| Body mass index | 0.05 | 0.98-1.13 | 0.16 |  |  |  |  |
| Smoking status | -0.81 | 0.18-1.08 | 0.45 |  |  |  |  |
| Hypertension status | 1.30 | 1.52-8.78 | <0.01 |  | 0.82 | 0.87-6.00 | 0.10 |
| Diabetic duration | 0.02 | 0.97-1.07 | 0.38 |  |  |  |  |
| Retinopathy | 1.53 | 2.04-10.57 | <0.01 |  | 1.66 | 2.05-13.57 | <0.01 |
| Insulin | 0.40 | 0.74-3.01 | 0.26 |  |  |  |  |
| Diuretics | 0.90 | 0.62-9.69 | 0.20 |  |  |  |  |
| ACEI/ARB | 0.28 | 0.67-2.67 | 0.44 |  |  |  |  |
| Triglycerides | 0.03 | 0.75-1.43 | 0.84 |  |  |  |  |
| Total Cholesterol | 0.22 | 0.94-1.66 | 0.13 |  |  |  |  |
| High-density lipoprotein | -0.60 | 0.22-1.38 | 0.20 |  |  |  |  |
| Low-density lipoprotein | 0.12 | 0.63-2.03 | 0.69 |  |  |  |  |
| HbA1c | 0.30 | 1.01-1.80 | 0.04 |  | 0.25 | 0.93-1.76 | 0.13 |

Abbreviations: Similar to Table 1

Table S3. Multivariable analysis for resting global longitudinal strain.

| Variables |  | Univariate |  |  |  | Multivariate |  |
| --- | --- | --- | --- | --- | --- | --- | --- |
|  | B | 95% CI | P |  | B | 95% CI | P |
| Age | 0.003 | -0.04-0.04 | 0.89 |  |  |  |  |
| Male | -0.10 | -0.84-0.64 | 0.79 |  |  |  |  |
| Body mass index | 0.14 | 0.07-0.21 | <0.01 |  | 0.10 | 0.03-0.18 | <0.01 |
| Smoking status | -0.40 | -1.33-0.52 | 0.39 |  |  |  |  |
| Hypertension status | 0.90 | 0.03-1.76 | 0.04 |  | 0.57 | -0.27-1.40 | 0.19 |
| Diabetic duration | 0.02 | -0.04-0.07 | 0.58 |  |  |  |  |
| Retinopathy | 1.02 | 0.25-1.79 | 0.01 |  | 0.81 | 0.01-1.61 | <0.05 |
| Insulin | 0.59 | -0.17-1.34 | 0.13 |  |  |  |  |
| Diuretics | 0.48 | -0.88-1.84 | 0.49 |  |  |  |  |
| ACEI /ARB | 0.28 | -0.48-1.05 | 0.47 |  |  |  |  |
| Triglycerides | 0.01 | -0.34-0.36 | 0.95 |  |  |  |  |
| Total Cholesterol | 0.31 | 0.03-0.58 | 0.03 |  | 0.15 | -0.13-0.43 | 0.29 |
| High-density lipoprotein | -1.61 | -2.55-(-0.68) | <0.01 |  | -0.95 | -1.91-0.02 | 0.05 |
| Low-density lipoprotein | -0.10 | -0.74-0.53 | 0.75 |  |  |  |  |
| HbA1c | 0.24 | -0.04-0.52 | <0.10 |  | 0.03 | -0.25-0.31 | 0.86 |

Abbreviations: Similar to Table 1

Table S4. Multivariable analysis for LV diastolic reserve function.

| Variables |  | Univariate |  |  |  | Multivariate |  |
| --- | --- | --- | --- | --- | --- | --- | --- |
|  | B | 95% CI | P |  | B | 95% CI | P |
| Age | 0.08 | 1.03-1.13 | <0.01 |  | 0.08 | 1.02-1.14 | <0.01 |
| Male | -0.99 | 0.16-0.87 | 0.02 |  | -0.94 | 0.14-1.01 | 0.08 |
| Body mass index | -0.03 | 0.89-1.06 | 0.97 |  |  |  |  |
| Smoking status | -0.10 | 0.33-2.50 | 0.85 |  |  |  |  |
| Hypertension status | 0.93 | 0.81-7.91 | 0.11 |  |  |  |  |
| Diabetic duration | 0.04 | 0.99-1.10 | 0.15 |  |  |  |  |
| Retinopathy | 1.22 | 1.48-7.79 | <0.01 |  | 1.07 | 1.04-8.17 | 0.04 |
| Insulin | 0.15 | 0.52-2.63 | 0.71 |  |  |  |  |
| Diuretics | -1.12 | 0.04-2.68 | 0.30 |  |  |  |  |
| ACEI /ARB | 0.50 | 0.71-3.79 | 0.25 |  |  |  |  |
| Triglycerides | 0.21 | 0.81-1.89 | 0.33 |  |  |  |  |
| Total Cholesterol | -0.51 | 0.36-1.00 | 0.05 |  | -0.36 | 0.40-1.22 | 0.21 |
| High-density lipoprotein | 0.90 | 0.86-7.09 | 0.09 |  | 0.41 | 0.35-6.54 | 0.59 |
| Low-density lipoprotein | 0.24 | 0.63-2.56 | 0.51 |  |  |  |  |
| HbA1c | 0.08 | 0.80-1.46 | 0.60 |  |  |  |  |

Abbreviations: Similar to Table 1

Table S5. Multivariable analysis for stress global longitudinal strain.

| Variables |  | Univariate |  |  |  | Multivariate |  |
| --- | --- | --- | --- | --- | --- | --- | --- |
|  | B | 95% CI | P |  | B | 95% CI | P |
| Age | 0.03 | -0.01-0.08 | 0.16 |  |  |  |  |
| Male | 0.10 | -0.80-0.99 | 0.83 |  |  |  |  |
| Body mass index | 0.08 | -0.01-0.17 | 0.07 |  | 0.08 | -0.02-0.17 | 0.10 |
| Smoking status | -0.40 | -1.53-0.74 | 0.49 |  |  |  |  |
| Hypertension status | 0.91 | -0.14-1.96 | 0.09 |  | 0.28 | -0.78-1.34 | 0.60 |
| Diabetic duration | 0.10 | -5.08-5.29 | 0.97 |  |  |  |  |
| Retinopathy | 2.22 | 1.35-3.10 | <0.01 |  | 2.15 | 1.14-3.16 | <0.01 |
| Insulin | 0.87 | -0.05-1.78 | 0.06 |  | 0.10 | -0.83-1.03 | 0.83 |
| Diuretics | 0.10 | -1.55-1.75 | 0.91 |  |  |  |  |
| ACEI /ARB | 0.52 | -0.40-1.44 | 0.27 |  |  |  |  |
| Triglycerides | -0.02 | -0.45-0.40 | 0.91 |  |  |  |  |
| Total Cholesterol | 0.21 | -0.14-0.55 | 0.24 |  |  |  |  |
| High-density lipoprotein | -1.22 | -2.40-(-0.04) | 0.04 |  | -0.74 | -1.92-0.44 | 0.22 |
| Low-density lipoprotein | -0.41 | -1.18-0.37 | 0.30 |  |  |  |  |
| HbA1c | 0.18 | -0.16-0.52 | 0.30 |  |  |  |  |

Abbreviations: Similar to Table 1
